# Whole brain radiotherapy (WBRT) after local treatment of brain metastases in melanoma patients: Statistical Analysis Plan

**DOI:** 10.1186/s13063-019-3555-5

**Published:** 2019-08-05

**Authors:** Serigne N. Lo, Angela M. Hong, Lauren E. Haydu, Tasnia Ahmed, Elizabeth J. Paton, Victoria Steel, George Hruby, Anh Tran, Rachael L. Morton, Anna K. Nowak, Janette L. Vardy, Katharine J. Drummond, Haryana M. Dhillon, Catherine Mandel, Richard A. Scolyer, Mark R. Middleton, Bryan H. Burmeister, John F. Thompson, Gerald B. Fogarty

**Affiliations:** 1 Melanoma Institute Australia, The University of Sydney, Sydney, NSW Australia; 2 Institute for Research and Medical Consultations (IRMC), Imam Abdulrahman Bin Faisal University, Dammam, Kingdom of Saudi Arabia; 3 Sydney Medical School, The University of Sydney, Sydney, NSW Australia; 4 Mater Hospital, North Sydney, NSW Australia; 5 Genesis Care, Mater Radiation Oncology, North Sydney, NSW Australia; 6 MD Anderson Cancer Center, Houston, TX USA; 7 Australia and New Zealand Melanoma Trials Group, North Sydney, NSW Australia; 8 Royal North Shore Hospital, St Leonards, NSW Australia; 9 NHMRC Clinical Trials Centre, The University of Sydney, Camperdown, NSW Australia; 10 Medical School, University of Western Australia, Perth, WA Australia; 11 Royal Melbourne Hospital, Parkville, VIC Australia; 12 Swinburne University of Technology, Melbourne, VIC Australia; 13 Royal Prince Alfred Hospital, Camperdown, NSW Australia; 14 University of Oxford, Oxford, UK; 15 Genesis Care Fraser Coast, Urraween, QLD Australia; 16 Genesis Care, Sydney, NSW Australia; 17 Department of Melanoma and Surgical Oncology, Royal Prince Alfred Hospital, Sydney, NSW Australia; 18 University of Technology Sydney, Sydney, NSW Australia; 19 St Vincent’s Hospital, Sydney, NSW Australia

**Keywords:** Radiotherapy, Metastases, Melanoma, Brain, Whole brain radiotherapy, Randomised trial

## Abstract

**Background:**

The WBRTMel trial is a multinational, open-label, phase III randomised controlled trial comparing whole brain radiotherapy (WBRT) to observation following local treatment of one to three melanoma brain metastases with surgery and/or stereotactic irradiation. The primary trial endpoint was to determine the effect of adding WBRT to local treatment on distant intracranial control, and the secondary endpoints were neurocognitive function, quality of life (QoL), performance status, overall survival, death from intracranial causes, death from melanoma and cost-effectiveness.

**Objective:**

The objective of this update is to outline and publish the pre-determined statistical analysis plan (SAP) before the database lock and the start of analysis.

**Methods:**

The SAP describes basic analysis principles, methods for dealing with a range of commonly encountered data analysis issues and the specific statistical procedures for analysing efficacy and safety outcomes. The SAP was approved after closure of recruitment and before completion of patient follow-up. It outlines the planned primary analyses and a range of subgroup and sensitivity analyses regarding the clinical and QoL outcomes. Health economic outcomes are not included in this plan but will be analysed separately. The SAP will be adhered to for the final data analysis of this trial to avoid analysis bias arising from knowledge of the data.

**Results:**

The resulting SAP is consistent with best practice and will allow open and transparent reporting.

**Conclusion:**

We have developed a SAP for the WBRTMel trial which will be followed to ensure high-quality standards of internal validity to minimise analysis bias.

**Trial registration:**

ANZ Clinical Trials Registry, ACTRN12607000512426. Registered on 9 October 2007. ClinicalTrials.gov, NCT01503827. Registered on 4 January 2012. Trial group reference numbers ANZMTG 01.07, TROG 08.05.

## Introduction

### Preface

Brain metastases are a common cause of death in patients with melanoma. The use of whole brain radiotherapy (WBRT) after excision and/or stereotactic radiosurgery (SRS) for melanoma brain metastases is variable because of the lack of high-quality evidence to guide practice. The trial’s rationale, feasibility and protocol were previously published [1–3]. In brief, the primary trial endpoint was to determine the effect of adding WBRT to local treatment on distant intracranial control, and the secondary endpoints were neurocognitive function (NCF), quality of life (QoL), performance status, overall survival, death from intracranial causes, death from melanoma and cost-effectiveness. In concordance with clinical trial requirements, the study was prospectively registered (ACTRN12607000512426 and NCT01503827). The trial completed the target accrual in September 2017, and the final participant will be followed up for at least 24 months.

### Purpose of the analyses

The statistical analyses will assess the clinical endpoints and safety of WBRT compared with the observation following excision and/or SRS.

## Study objectives and endpoints

### Study objectives

This trial aims to improve the treatment of brain metastases for patients with stage IV melanoma by using WBRT to improve disease control and maintain cognitive performance and QoL.

### Endpoints

#### Primary endpoint

The primary endpoint of the study is defined as distant intracranial failure within 12 months of randomisation. Distant intracranial failure is evaluated through magnetic resonance imaging (MRI) assessment, as reported by participating institutions, and is defined as new lesions appearing 1 cm or more from previous index metastases, coded as a binary outcome of success or failure.

#### Key secondary endpoint

The main NCF endpoint is decline in the mean raw score of the Hopkins Verbal Learning Test-Revised (HVLT-R) with Delayed Recall at 4 months, using (1) relative change defined as ΔHVLT = 100*(HVLT_B_ – HVLT_F_) ÷ HVLT_B_, where indexes B = baseline and F = a pre-specified follow-up time point [4], and (2) Reliable Change Index (RCI) defined as a decline of ≥ 5 points from baseline [5].

#### Secondary endpoints

Several secondary endpoints are assessed during the study.

- *Neurocognitive decline* will be computed for HVLT-R (Total Recall, Delayed Recall, Delayed Recognition), Controlled Oral Word Association Test, Trail Making Test Parts A and B, Stroop Color and Word Test (Adult Version, Stroop Color, Stroop Word, Stroop Color-Word, Stroop Interference), and Digit Span (total score) [6, 7].
- *Time to cognitive failure* is defined as the time from randomisation to date of first cognitive failure on any of the NCF tests or death for patients who die due to an intracranial cause prior to assessment or NCF for patients who were alive but could not complete the neurocognitive test due to neurological disability. Cognitive failure for patients with NCF assessment scores will be analysed using both raw and standardised scores and defined by a significant change in the RCI. Patients without neurognitive failure are censored at their last date of contact.
- *Distant intracranial failure within 3, 6 and 9 months of follow-up* and over the entire study period

(see primary endpoint definition)

- *Time to distant intracranial failure as determined by MRI*

This is defined as the time to recurrence of disease to a distance of 1 cm or more from previously treated metastases from the date of randomisation. It is measured by the time difference between the randomisation MRI and intracranial failure MRI identifying recurrence.

- *Time to local intracranial failure as determined by MRI*

This is defined as the time to recurrence of disease within 1 cm of previously treated metastases from the date of randomisation. It is measured by the time difference between the randomisation MRI and intracranial failure MRI identifying recurrence. Local intracranial failure rates within 3, 6, 9 and 12 months of follow-up and over the entire study period will also be computed.

- *Time to overall (first distant or local) intracranial failure*

This is determined through MRI from observable deterioration and is defined as the time to the first intracranial recurrence of disease anywhere (distant or local) from randomisation. Overall intracranial failure rates within 3, 6, 9 and 12 months of follow-up and over the entire study period will also be computed.

- *Time to deterioration in performance status as measured by the Eastern Cooperative Oncology Group (ECOG) Performance Status*

This is defined as the time that elapses between randomisation and the first recorded worsening in ECOG Performance Status. This increase is measured from the randomisation visit ECOG Performance Status score.

- *QoL outcomes*

The outcomes are measured using the European Organisation for the Research and Treatment of Cancer (EORTC) Quality of Life Questionnaires (QLQs), namely:

- EORTC QLQ-C30: This questionnaire has 30 items arranged into nine scales and six single items. The scales are divided into five function scales (physical, role, cognitive, emotional, social function), three symptom scales (fatigue, pain, nausea or vomiting) and one global health status/QoL scale [8].The six single items address specific symptoms: dyspnea, appetite loss, insomnia, constipation and diarrhoea, with one question addressing the financial impact of the disease.
- EORTC QLQ-BN20 – Brain Cancer Module: This questionnaire contains 20 items organised into four scales: future uncertainty (four items), visual disorder (three items), motor dysfunction (three items) and communication deficit (three items), and seven single items (headaches, seizures, drowsiness, hair loss, itchy skin, weakness of legs, bladder control) [9]
- EORTC EQ-5D-5 L: This consists of two questionnaire systems: (1) the EuroQol visual analogue scale (EQ VAS), which records the respondent’s self-rated health on a 20-cm vertical, visual analogue scale with endpoints labelled ‘Best health you can imagine’ and ‘Worst health you can imagine’ [10]; (2) the five dimensions questionnaire (mobility, self-care, usual activities, pain/discomfort and anxiety/depression), where each dimension has five levels (no problems, slight problems, moderate problems, severe problems and extreme problems).

In addition to associated dimension/items used as QoL outcomes, time to deterioration in health-related QoL parameters will also be derived and compared between groups. The deterioration in health-related QoL outcomes will be measured by the EORTC QLQ-C30 and QLQ-BN20 questionnaires. The primary QoL endpoint will be time to deterioration in role function from randomisation, with deterioration defined as a decrease of ≥10 points on a 0–100 scale persisting for at least 4 weeks. Secondary endpoints will be time to deterioration in global QoL, drowsiness, communication difficulties, motor dysfunction and social function items/domains.

- *Survival endpoints*

Two sets of survival endpoints are considered: (1) overall survival defined as time to death due to any cause and (2) disease-specific outcomes which include time to death due to intracranial disease and other melanoma-specific cause. Survival time is assessed from date of randomisation to that of death. Patients remaining alive or lost to follow-up will be censored at the date of last contact.

- *Neurocognitive assessments*

The *Global Deficit Score (GDS)* derived from NCF tests at each assessment point [11]. Cognitive impairment is defined as a GDS of ≥ 0.5 [12].

#### Tertiary endpoints

Although not statistically powered for these endpoints, this trial will provide a unique opportunity to assess observation vs. hippocampal avoidance (HA-WBRT) vs. WBRT in a prospective fashion for NCF outcomes. In particular, this analysis will add to the ongoing debate on the impact of WBRT on NCF and intracranial control [13, 14].

The following variables will be considered:

- Mean relative NCF change from baseline at 2, 4, 6 and 12 months based on:
  - HVLT-R Delayed Recall
  - HVLT-R Total Recall
  - HVLT-R Delayed Recognition.
- Global cognitive deterioration survival based on the definition of cognitive impairment of 1 standard deviation (SD) in at least one of the following cognitive tests: HVLT-R, Trail Making Test or Controlled Oral Word Association.

The following definitions of cognitive impairment will also be explored:

- ○ 2 SD decline in at least one cognitive test
- ○ 1 SD decline in at least two cognitive tests
- ○ 2 SD decline in at least two cognitive tests.

### Safety outcomes

Adverse events (AEs) and any AE reported as a serious adverse event (SAE) will be included in this analysis. An AE is any adverse change (developing or worsening) from the patient’s pre-treatment condition, including intercurrent illness. AEs are to be recorded at the baseline visit and then every 2 months at the follow-up visits or until the patient experiences distant intracranial failure.

In this trial an SAE is defined as any untoward medical occurrence which occurs during or within 90 days of randomisation and which:

1. Results in death
2. Is life-threatening
3. Requires in-patient hospitalisation or prolongation of existing hospitalisation
4. Results in persistent or significant disability/incapacity
5. Results in a congenital anomaly/birth defect
6. Is a medically important event or reaction
7. Is a grade 3 (National Cancer Institute [NCI] Common Terminology Criteria for Adverse Events [CTCAE] v4.03) toxicity
8. Could be related to radiation therapy.

The NCI CTCAE v4.03 was used to classify and grade the intensity of AEs/SAEs and their relationship to study treatment.

## Study methods

### General study design and plan

**Fig. 1:**
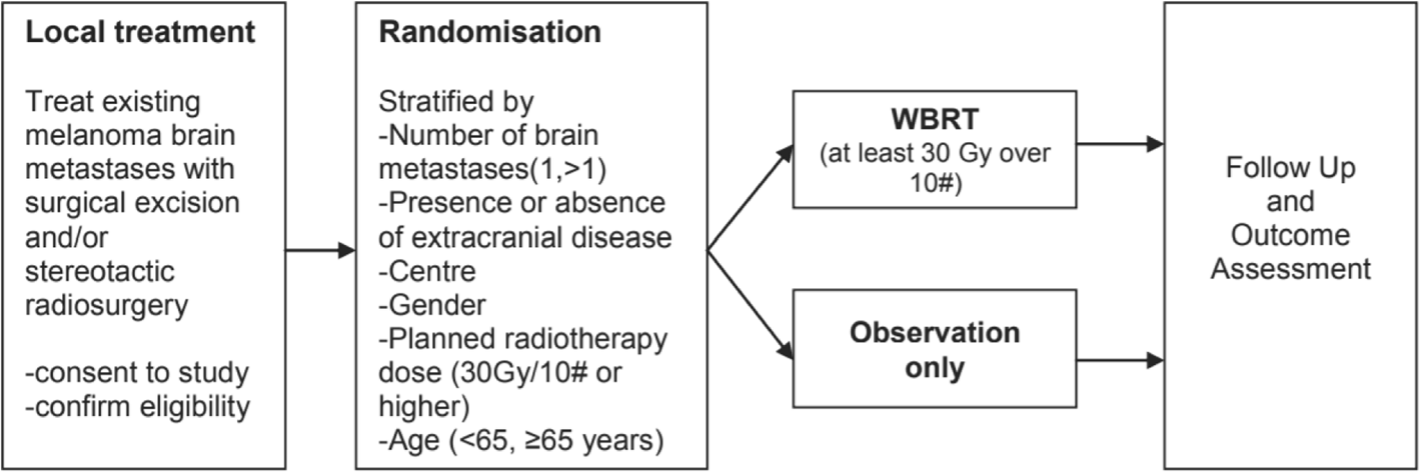
Trial schema

This is a multinational, open-label, stratified, 2-arm parallel phase III trial. Figure 1 shows the schema for the trial.

### Inclusion and exclusion criteria and general study population

#### Patient population

Patients were recruited from participating multidisciplinary melanoma centres. Patients were identified via routine scanning demonstrating asymptomatic metastases or following investigation of intracranial symptoms.

#### Inclusion criteria

Patients were included in the study only if they met all of the following criteria:

1. One to three (1–3) intracranial metastases on MRI from melanoma, locally treated with either surgical excision and/or SRS. It was assumed that the metastases were melanoma if the patient had documented histological concurrent extracranial disease that had already made the patient stage IV. If the cerebral lesion(s) were the first presentation of stage IV disease, then one metastasis had to be histologically proven to be melanoma for the patient to be included in the study.
2. Had a life expectancy of at least 6 months.
3. Aged 18 years or older.
4. WBRT must have been able to commence within 8 weeks of completion of local treatment and within 4 weeks of randomisation.
5. Able to have an MRI brain scan with contrast. Estimated glomerular filtration rate (eGFR) was adequate at the discretion of the radiologist and capable of having gadolinium-containing contrast medium for MRI (as per practice guidelines).
6. Completion of local treatment of all these metastases no more than 6 weeks prior to randomisation.
7. An ECOG Performance Status between 0 and 2 at randomisation.
8. Computed tomography (CT) or positron emission tomography (PET) scan of chest, abdomen and pelvis as a minimum prior to randomisation. Scans must have been taken within 12 weeks of randomisation.
9. Serum lactate dehydrogenase (LDH) level must have been ≤2× upper limit of normal.
10. Was able to provide written informed consent.

#### Exclusion criteria

Patients were excluded from the study for any of the following reasons:

1. Any untreated intracranial disease
2. Any previous intracranial treatment (surgical excision and/or stereotactic irradiation treatment and/or WBRT) prior to this diagnosis of intracranial melanoma
3. Evidence of leptomeningeal disease on pre-local treatment MRI scan
4. Patients with prior cancers, except for those:
  - Diagnosed more than 5 years ago with no evidence of disease recurrence within this time
  - With successfully treated basal cell or squamous cell skin carcinoma
  - With carcinoma in situ of the cervix
5. With a medical or psychiatric condition that compromises ability to give informed consent or complete the protocol
6. (Women of childbearing potential) with a positive urine pregnancy test (within 7 days of randomisation into the trial).

In order to be eligible for the main study, patients must have cleared all of the preceding inclusion and exclusion criteria. If centres had access to intensity-modulated radiotherapy (IMRT) for patients with brain metastasis, randomisation could occur before local radiotherapy treatment to allow for the use of simultaneous integrated boost during WBRT (see the Interim analyses and data monitoring section). In addition, patients were excluded from the neurocognitive and QoL aspects of the study if their fluency of (oral and written) English was less than year 8 standard.

### Randomisation and blinding

Patients were randomised at a 1:1 ratio using an interactive voice response (randomisation) system (IVRS). Randomisation was stratified by the following factors:

- Number of cerebral metastases (1 only vs. > 1)
- Presence or absence of extracranial disease (seen on clinical review and/or imaging)
- Centre
- Sex (male vs. female)
- Planned whole brain radiotherapy dose, 30 Gray in 10 fractions (30 Gy/10#) or higher
- Age (< 65 or ≥ 65 years old)

### Study variables

All parameters and their timing of collection are summarised in Table 1.

**Table 1:** Table 1 Schedule of study parameters and collection: months 0–24 and beyond 24 months

	Pre-randomisation	Baseline		Months 0–24 Follow-up visit every 2 months (± 2 weeks); MRI every 3 months (± 4 weeks)																					Intracranial failure
				2	3	4		6	8	9		10	12	14		15	16	18	20	21		22	24		
Initial local treatment and confirmation of melanoma	X																								
Urine pregnancy test		X																							
eGFR	X																								
MRI of brain	X	X			X			X		X			X			X		X		X			X		X
Disease status^a^	X	Any imaging after randomisation will be as clinically indicated by local protocol or for suspicion of systemic progression..																							
ECOG Performance Status	X			X		X		X	X			X	X	X			X	X	X			X	X		X
AE/SAE reporting		X		X		X		X	X			X	X	X			X	X	X			X	X		X
Medical history		X																							
Physical examination		X		X		X		X	X			X	X	X			X	X	X			X	X		X
Systemic therapies		X		X		X		X	X			X	X	X			X	X	X			X	X		X
Steroid use		X		X		X		X	X			X	X	X			X	X	X			X	X		X
Participation in other clinical trials		X		X		X		X	X			X	X	X			X	X	X			X	X		X
QoL questionnaires		X		X		X		X	X			X	X	X			X	X	X			X	X		X
Health economic resource use questionnaires		X		X		X		X	X			X	X	X			X	X	X			X	X		X
NCF assessments		X		X		X		X	X			X	X	X			X	X	X			X	X		X
WBRT	For patients randomised to the WBRT treatment arm: WBRT is to commence < 4 weeks from randomisation and no more than 8 weeks from completion of local brain metastases treatment For patients who receive salvage WBRT during follow-up: Patients may undergo salvage WBRT at any time during the observation period, at the local investigator’s discretion																								
	Beyond 24 months^b^																								
	Patients who have experienced intracranial failure by 24 months Follow-up visit every 2 months (± 2 weeks); MRI every 3 months (± 4 weeks)																Patients who have *NOT* had an intracranial failure by 24 months Follow-up visit every 3 months (± 2 weeks); MRI every 6 months (± 4 weeks)								Intracranial failure
	26	27	28	30	32	33	34	36	38	39	40	42	44	45	46	48	27	30	33	36	39	42	45	48	
MRI of brain		X		X		X		X		X		X		X		X		X		X		X		X	X
Disease status^a^	Any imaging after randomisation will be as clinically indicated by local protocol or for suspicion of systemic progression																								
ECOG Performance Status	X		X	X	X		X	X	X		X	X	X		X	X	X	X	X	X	X	X	X	X	X
AE/SAE reporting	X		X	X	X		X	X	X		X	X	X		X	X	X	X	X	X	X	X	X	X	X
Physical examination	X		X	X	X		X	X	X		X	X	X		X	X	X	X	X	X	X	X	X	X	X
Systemic therapies	X		X	X	X		X	X	X		X	X	X		X	X	X	X	X	X	X	X	X	X	X
Steroid use	X		X	X	X		X	X	X		X	X	X		X	X	X	X	X	X	X	X	X	X	X
Participation in other clinical trials	X		X	X	X		X	X	X		X	X	X		X	X	X	X	X	X	X	X	X	X	X
QoL questionnaires	X		X	X	X		X	X	X		X	X	X		X	X	X	X	X	X	X	X	X	X	X
Health economic resource use questionnaires	X		X	X	X		X	X	X		X	X	X		X	X	X	X	X	X	X	X	X	X	X
NCF assessments	X		X	X	X		X	X	X		X	X	X		X	X	X	X	X	X	X	X	X	X	X
WBRT	For patients randomised to the WBRT treatment arm: WBRT is to commence < 4 weeks from randomisation and no more than 8 weeks from completion of local brain metastases treatment For patients who receive salvage WBRT during follow-up: Patients may undergo salvage WBRT at any time during the observation period, at the local investigator’s discretion																								

^a^ Disease status assessed by staging CT or PET scans

^b^ Follow-up visits will continue beyond month 48 and until death

## Sample size

Assuming the proportion of patients having distant intracranial metastases at 12 months post-randomisation would be 55% in the local control arm (surgery and/or SRS) and 33% in the WBRT arm, a total of 220 patients provided more than 84% power to detect the absolute reduction of 22%, allowing for up to 10% non-compliance and a two-sided alpha of 5%.

The initial sample size was based on a total sample size of 200 to achieve 80% to reach the study primary objective. However, modern radiation therapy technologies to spare the hippocampus during WBRT became available in some centres during the study. In addition, phase 2 data showed that HA-WBRT was superior in terms of NCF outcomes, a secondary but important endpoint of the study. The investigators felt that if the option of HA-WBRT was not included, accrual would suffer. Therefore, the sample size was increased by protocol amendment from the initial 200 to 220 to enable the recruitment of a total of 27 patients treated with HA-WBRT. A sample of 27 patients treated with HA-WBRT would achieve 80% power to detect a mean relative decline ≤7.5% against 30% mean relative decline in a historical control from baseline to 4 months. The sample size calculation assumed an estimated SD of 0.41, and the level of significance (alpha) was set to 5% using a one-sided Wilcoxon test assuming that the actual distribution is normal.

At the time of the protocol amendment, 27 January 2016, 190 patients were enrolled in the study; among them 16 had HA-WBRT. Therefore, 11 extra HA-WBRT patients were needed. We expected to accrue these additional 11 HA-WBRT patients by enrolling a further 30 patients, bringing the total sample size to 220.

## General considerations

The analysis principles are as follows:

1. All analyses will be conducted on an intention-to-treat basis.
2. All randomised patients will be analysed in the group to which they were assigned regardless of protocol violations. The only exception will be patients whose consent to use their data in the analysis is withheld or withdrawn.
3. All tests will be two-sided with a nominal level of alpha of 5%.
4. Treatment effect estimated as difference in means, odds ratio, hazard ratio or subdistribution hazard and their 95% confidence interval (CI) will be reported for all outcomes.
5. Adjusted analyses including the stratification factors will be performed as sensitivity analyses.
6. Subgroup analyses will be carried out irrespective of whether there is a significant effect of treatment on outcome.
7. *P* values will not be adjusted for multiplicity. However, the outcomes are clearly categorised by degree of importance (primary to tertiary), and a limited number of subgroup analyses are pre-specified.
8. All summary tables will be annotated with the total population size relevant to each treatment group.
9. *P* values ≥ 0.001 will be reported to three decimal places; *P* values less than 0.001 will be reported as ’< 0.001’. The mean, SD and any other statistics other than quantiles will be reported to one decimal place greater than the original data. Quantiles, such as median, or minimum and maximum will use the same number of decimal places as the original data. Estimated parameters, not on the same scale as raw observations (e.g. regression coefficients), will be reported to three significant figures.
10. Analyses will be conducted primarily using SAS, version 9.4 or later and R 3.4.1 or later.

### Analysis population

The final analysis will include all patients who were randomised and have all baseline assessments performed.

### Subgroups

The primary endpoint and the secondary endpoints (excluding the key secondary endpoint) will be assessed for the following subgroups:

- One vs. more than one cerebral metastasis
- Presence of extracranial disease vs. none
- Patients aged < 65 years of age or ≥ 65 years of age
- Sex: male vs. female
- Treated with systemic therapy (No vs. prior to randomisation vs. within 12 months after randomisation)
- Treated with steroid (No vs. prior to randomisation vs. within 12 months after randomisation)

### Interim analyses and data monitoring

An independent Data and Safety Monitoring Committee (DSMC) was established to monitor the occurrence of serious clinical and biological events. The DSMC reviewed unblinded data to examine patient characteristics, treatment compliance, outcomes and safety events once the first 100 patients had completed 12 months of follow-up. Only DSMC members and statisticians compiling closed-session reports for board meetings have had access to unblinded interim data and results. The Haybittle–Peto boundary was used as a stopping guideline for efficacy to maintain an overall type I error rate of 5% level [15]. The incidence of distant intracranial failure over time was calculated using a risk analysis accounting for extracranial melanoma progression and death from any cause other than intracranial disease as competing risks. Distant intracranial failure and death from intracranial disease were counted as events. After the interim review, the DSMC made the following recommendation in the closed minutes: *The quality of the data collection for the major endpoints of this study were meeting an excellent standard, the pre-specified stopping criteria have not been met, and recommended continuation of the study*.

### Multinational multicentre study

This is a trial involving three countries (Australia, Norway and the UK) in which 24 centres have contributed data for at least one patient. Although study centres were included as a stratification factor in the randomisation scheme, analyses of primary and secondary efficacy outcomes will not adjust for study centre, because it is anticipated some centres may be too small and may not contribute any events to the pooled data. However, if a positive treatment effect on the primary composite outcome or the component events emerges, study centres will be combined by country (Australia, Norway and the UK) to assess the homogeneity of the treatment effect across countries. Potential heterogeneity will be tested by including a treatment-by-country interaction term in a multivariate model with treatment and country as the main effects.

## Data analysis

### Demographic and baseline variables

A description of the following baseline characteristics will be presented by treatment group. All continuous variables will be summarised using the following descriptive statistics: *n* (non-missing sample size), mean (SD), median (minimum – maximum). The frequency and percentages (based on the non-missing sample size) of observed levels will be reported for all categorical measures.

The baseline measures for all patients are the following:

1. Sex
2. Age (years)
3. Country of birth
4. Main language spoken at home
5. Indigenous status
6. Country of residence
7. Date primary melanoma diagnosed
8. Primary melanoma has been treated
9. Primary melanoma present at the time of randomisation
10. Type of primary melanoma (cutaneous, mucosal, choroidal, other)
11. Primary melanoma site (for cutaneous tumour)
12. Breslow thickness
13. Mitotic rate
14. Ulceration
15. Satellite
16. Desmoplasia
17. Number of intracranial metastases at baseline
18. Number of extracranial disease sites at randomisation
19. Planned total dose of whole brain radiotherapy
20. Number of years of education completed
21. Wide Range Achievement Test (WRAT) score
22. Work status at time of melanoma diagnosis
23. Reason if not working
24. Indicate level of income
25. Indicate source of that income
26. Health insurance status of the patient

*Medical history*

27. Local recurrence diagnosed in the past
28. Previous local recurrence treated
29. Local recurrence present at baseline
30. Regional recurrence diagnosed in the past
31. Previous regional recurrence treated
32. Regional recurrence present at baseline
33. Distant recurrence diagnosed in the past
34. Number of distant recurrences
35. Organ affected by distant disease
36. Surgery performed on the distant disease
37. Radiotherapy given at the site of distant disease

*Intracranial disease at baseline*

38. Intracranial disease present at baseline
39. Location(s) of intracranial lesion
40. Maximum dimension of lesions(s); Diameter 1
41. Maximum dimension of lesion(s); Diameter 2
42. Lesion 1
  - Neurosurgery performed
  - Excision completed
  - Histopathology performed on the lesion
  - Melanoma confirmed by haematoxylin and eosin (H&E) staining
  - Melanoma confirmed by S100 staining
  - Melanoma confirmed by Human Melanoma Black (HMB45) staining
  - Lesion treated with stereotactic irradiation
  - Diameter of stereotactic volume treated (millimetres)
  - Dose of lesion (Grays)
43. Lesion 2
  - Neurosurgery performed
  - Excision completed
  - Histopathology performed on the lesion
  - Melanoma confirmed by H&E staining
  - Melanoma confirmed by S100 staining
  - Melanoma confirmed by HMB45 staining
  - Lesion treated with stereotactic irradiation
  - Diameter of stereotactic volume treated (millimetres)
  - Dose of lesion (Grays)
44. Lesion 3
  - Neurosurgery performed
  - Excision completed
  - Histopathology performed on the lesion
  - Melanoma confirmed by H&E staining
  - Melanoma confirmed by S100 staining
  - Melanoma confirmed by HMB45 staining
  - Lesion treated with stereotactic irradiation
  - Diameter of stereotactic volume treated (millimetres)
  - Dose of lesion (Grays)

### Process measurement

The summary statistics will be produced in accordance with the Demographic and baseline variables section by follow-up visit (baseline, 2, 4, 6, 8, 10, 12, 14, 16, 18, 20, 22, 24 months). Differences between groups at each follow-up visit will be assessed using the mean difference and its 95% CI for continuous variables and the odds ratio along with the corresponding 95% CI for categorical variables. The overall treatment effect will be estimated using repeated measures mixed linear model (MLM) analysis or generalised estimating equation (GEE) methods as appropriate. The following variables will be summarised:

1. Physical examination (ECOG Performance Status)
2. Systemic therapies use
3. Steroid use

### Treatment compliance

The assessment of treatment compliance in the WBRT group includes:

1. Major variations
  - Duration of radiotherapy treatment
  - Total dose of WBRT
2. Minor variations
  - Duration from randomisation to radiotherapy
  - Maximum clinical radiotherapy dose to brain CT venography (CTV)
  - Minimum dose received

Each variable will be summarised by frequency and percentages.

### Efficacy analyses

Efficacy analyses will be conducted on the basis of ’intention to treat’ and will be stratified by treatment group. Efficacy analysis will be adjusted with the four stratification factors (one vs. more than one cerebral metastasis, presence of extracranial disease vs. none, patients aged < 65 years of age or ≥ 65 years of age, sex: male vs. female). All group comparisons will be two-tailed with a 5% significance level. For efficacy time-to-event outcomes, patients with incomplete follow-up who did not experience a relevant outcome event will be censored at the time of their last contact (that is, their time data will contribute to analyses).

#### Primary outcome

The primary endpoint, defined as distant intracranial failure within 12 months of follow-up (binary failure/success), will be compared using a Pearson chi-squared test. In cases where the expected count per cell is less than 5, Fisher’s exact test will be used. Overall treatment effect will be summarised using the odds ratio and its 95% CI). Absolute treatment effect (risk difference) will also be computed. Treatment success was defined as patients who did not experience distant intracranial failure within 12 months after starting treatment. Patients who died prior to the 12 months assessment without any distant intracranial failure sign will also be considered as a success. In addition, patients who experienced local failure but died without clinical evidence of distant failure will be recorded as a success. When patients have withdrawn consent or have been lost to follow-up before the 12 months assessment, the last follow-up intracranial assessment status will be used for the primary endpoint analysis.

#### Key secondary outcomes

Neurocognitive endpoint is defined as decline in the mean raw score of the Hopkins Verbal Learning Test-Revised (HVLT-R) Delayed Recall at 4 months, using mean relative change and Reliable Change Index (RCI).

The Global Deficit Score (GDS) derived from NCF tests at each assessment point where cognitive impairment is defined as a GDS of ≥0.5. The proportion of patients with cognitive impairment in each group (Observation, HA-WBRT and WBRT) will be summarised by frequency and rate with its two-sided 95% Clopper–Pearson exact CI. The denominator will be defined as the total number of patients for whom the GDS is computed. No formal inference will be performed given the study is not designed for a formal comparison between groups. Table 2 shows the calculation of GDS.

**Table 2:** Calculation of GDS

Cognitive domain	Test and output	Calculation
Memory	Hopkins Verbal Learning Test: A) Total Recall B) Delayed Rrecall C) Delayed Recognition	1 = (A + B + C)/3
Verbal fluency	Controlled Oral Word Association: A) Total Letter Fluency B) Category Fluency C) Written Fluency	2 = (A + B + C)/3
Information processing	Trail Making Test A	3
Attention	Digit Span total score	4
Executive function	Trail Making Test B	5
	Stroop Color and Word:	6 = (A + B + C + D)/4
	A) Stroop Color	
	B) Stroop Word	
	C) Stroop Color-Word	
	D) Stroop Interference	7 = (5 + 6)/2
Total GDS		8 = (1 + 2 + 3 + 4 + 7)/5

#### Secondary outcomes

We will analyse distant intracranial failure at 3, 6 and 9 months and over the entire study follow-up period, and local intracranial failure and overall intracranial failure at 3, 6, 9 and 12 months and over the entire study follow-up period. Analysis will be produced in accordance with the primary outcome.

##### Time-to-event outcomes

All secondary time-to-event outcomes with the exception of overall survival and time to deterioration in performance status will be analysed in the context of the competing risks [16]. For instance, death from any other cause than intracranial disease will prevent observation of a distant intracranial event. Therefore, the incidence of distant intracranial failure over time will be calculated using risk analysis accounting for death from any cause other than intracranial disease as a competing risk. The incidence of intracranial events between the treatment and observation arms will be compared using Gray’s test for equality while accounting for competing risks (non-intracranial death) [17]. Other time-to-event endpoints include time to local intracranial failure (competing with both death or distant failure as local failure is captured only before distant failure), time to overall (first distant or local) intracranial failure (competing with death), time to melanoma death (competing with non-melanoma death) and time to intracranial death (competing with other causes of death). These endpoints will also be analysed using a competing risks approach.

In addition, all time-to-event outcomes will be summarised graphically over the entire study follow-up period through (1) Kaplan–Meier methods with log-rank test stratified with the four stratification factors to assess the differences between treatment arms and (2) a cumulative incidence function for competing risk outcomes, with Gray’s test for differences between treatment arms. Hazard ratios using the Cox proportional hazard model stratified with the four stratification factors, or subdistribution hazard ratios using the Fine and Gray model adjusted with the four stratification factors and their associated 95% CIs will be displayed in corresponding figures as appropriate.

##### Overall survival

The outcome will be described using the product limit method of Kaplan–Meier. Survival curve distribution differences between treatment groups will be tested using the log-rank test. Hazard ratios and corresponding 95% CIs will be computed using the Cox proportional hazard model. The validity of the proportional hazards assumption will be tested using Schoenfeld residuals plots and corresponding test statistics. In contrast, if the proportional hazard assumption is violated (i.e. the hazard ratio is changing over time), an alternative robust measure known as restricted mean survival time (RMST), which does not rely on a specific assumption, will be generated in each arm. Then instead of hazard ratio, the difference in RMST along with its 95% CI will be computed to evaluate the potential benefit of WBRT effects over the study duration.

##### Time to deterioration in performance status

The analysis will be produced in accordance with the overall survival outcome analysis.

##### Time to deterioration in health-related quality of life (QoL) parameters

This outcome is measured by EORTC QLQ-C30 with Brain Cancer Module (EORTC QLQ-BN20) questionnaires. The completion rates for quality of life questionnaires (QLQs) at the baseline visit and at each 2 monthly follow-up visit will be determined for each treatment group together with descriptive summaries of scores. The cumulative incidence function will be used to describe patterns of deterioration in health-related QoL. A significant difference between groups will be tested using Gray’s test.

##### QoL outcomes

Utility-based QoL will be derived from QLQ-C30 using the McKenzie mapping algorithm. EQ-5D-5 L utilities will be calculated using the Australian tariff [18]. The continuous utility endpoints will be summarised by mean (SD) and stratified by assessment time points (baseline, 2–48 months) and by treatment group [19]. The mean difference between the two arms and their corresponding 95% CI will be provided at each assessment time point. The average effect of the intervention on each continuous outcome, over the entire study period, will be estimated through an MLM with random intercept, adjusting with associated baseline score, time (categorical) and interaction between treatment and time. In addition, an estimate of the intervention effect adjusted for the four stratification factors (one vs. more than one cerebral metastasis, presence of extracranial disease vs. none, patients age < 65 vs. ≥ 65 years old, and sex: male vs. female) will also be reported.

Categorical dimension/items will be analysed using Likert-type scales and stratified by treatment group.

##### Deterioration in neurocognitive function (NCF)

The main NCF endpoint will be defined as a decline in the mean score of the HVLT-R Delayed Recall at pre-specified follow-up visits. The proportion of patients completing NCF assessments at the baseline visit and at each 2 monthly follow-up visit will be determined for each of the treatment groups (WBRT and Observation) together with a descriptive summary of raw, standardised Z-scores, a composite Z-score and GDS scores. The summary statistics will be produced in accordance with the Process measurement section by follow-up visit (2, 4, 6, 8, 10 and 12 months). These will be standardised adjusting for age, education and gender, and analysed for change from baseline to pre-specified assessment visit. Decline will be defined using the RCI [6, 7] and will also be compared between the arms. Mean score changes and 95% CI will also be represented graphically from baseline over time. The overall effect of the intervention on each continuous NCF variable will be estimated through an MLM with random intercept, adjusting with associated baseline score, time (categorical) and also interaction between treatment and time. Overall effect for categorical NCF variables will be estimated using GEE regression with a log-binomial link. The following variables will be summarised:

1. HVLT
2. Controlled Oral Word Association Test
3. Trail Making Test Parts A and B
4. Stroop Color and Word Test (Adult Version)
5. Digit Span.

All analyses will be based on available data (complete case). However, if more than 10% of the data are missing for an outcome, a multiple imputation method will be carried out as a sensitivity method assuming data are missing at random (MAR). The MAR assumption indicates that the missingness mechanism depends on the observed outcome but not on the missing outcome, which seems an appropriate assumption in this study [20].

Exploratory analyses will be conducted adjusting for prognostic factors. Other time-to-event endpoints will also be analysed using similar methods. Time to deterioration in NCF will be analysed using both raw and standardised scores and defined by a significant change in the RCI (decline of ≥ 5 points from baseline). Outcomes will be analysed using a competing approach assuming deterioration in NCF is competing with any failure (local/distant intracranial failure or death).

### Exploratory efficacy analyses

#### Subgroup analysis

The subgroup analyses will assess potential differences in treatment effects, through an interaction effect. Evidence of heterogeneity of treatment effects among subgroups will be demonstrated by the level of statistical significance of the interaction term between treatment group and subgroup using either a logistic regression (for a binary outcome) or Cox regression (Overall survival) using a threshold of significance of 0.05. The subgroup analyses will remain exploratory and hypothesis-generating given the study is not specifically powered to test any subgroup. Results will be presented as forest plots with *P* values for heterogeneity (interaction test) for each pair of subgroups displayed.

#### Tertiary endpoints

Exploratory analysis will be performed by simultaneously comparing Observation vs. HA-WBRT vs. WBRT on the variables listed below. Time-to-event outcomes will be analysed in accordance with the Efficacy Analyses section using a competing risks model or the Cox proportional hazard model as appropriate. The cumulative incidence function or Kaplan–Meier survival curves will be provided as appropriate. Test of difference between the three groups will be performed using one-way analysis of covariance (ANCOVA) for continuous variables. For categorical variables, GEE regression with a log-binomial link will be used. All models will be adjusted with their baseline measurements and will include the following variables:

1. Global cognitive deterioration survival
2. Time to local intracranial failure
3. Time to distant intracranial failure
4. Time to overall survival
5. Deterioration in NCF measured as a significant deterioration compared with baseline (5-point drop) at 2, 4, 6, 8, 10 and 12 months in:
  - HVLT-R Delayed Recall
  - HVLT-R Total Recall
  - HVLT-R Delayed Recognition.

Deterioration in NCF will be represented graphically from baseline over time.

The preceding analysis will be replicated by the stratification factors: one vs. more than one cerebral metastasis, presence of extracranial disease vs. none, < 65 years vs. ≥ 65 years of age, and sex: male vs. female.

### Safety analyses

Safety outcomes will be analysed via frequencies for the number of episodes of adverse events (AEs) and via frequencies and percentages for the number of patients with at least one episode per cohort (with the denominator being the total number of patients in each group).

All AEs (graded per Common Terminology Criteria for Adverse Events [CTCAE]), even those not analysed, will be reported stratified by treatment arm.

## Conclusion

We have developed a statistical analysis plan (SAP) for the ANZMTG 01.07 WBRTMel study. This plan will be followed to ensure high-quality standards of internal validity to minimise analysis bias. The SAP was approved and signed off by the Trial Management Committee on 1 October 2018. Following data integrity checks of the primary and secondary 12 months outcomes, the statistical analysis specified in the SAP was started January 2019.

## List of tables and figures

A list of proposed tables and a list of proposed figures are displayed in this section. Reporting of the trial results is based on the International Conference on Harmonisation of Technical Requirements for Registration of Pharmaceuticals for Human Use (ICH) guideline for reporting clinical trial results [21].

### Tables

- (1) Patient characteristics stratified by treatment arm
- (2) Medical history by treatment arm
- (3) Distant disease at baseline by treatment arm
- (4) Lesions summary (stratified by Lesion 1, Lesion 2, Lesion 3, etc.) by treatment arm
- (5) Physical examination by treatment arm
- (6) Systemic therapy and steroid use over treatment by treatment arm
- (7) Treatment compliance by treatment arm
- (8) Primary and secondary distant intracranial failure by treatment arm
- (9) Key secondary outcome
- (10) The EuroQol visual analogue scale (EQ VAS)
- (11) Deterioration in NCF over time by treatment arm
- (12) Subgroup analysis (replicate Tables (8) and (10) for each subgroup as defined in Subgroups section)
- (13) Tertiary outcomes (replicate Tables (1) (8) and (9), stratified by Observation vs. HA-WBRT vs. WBRT)
- (14) AE and SAE summary by treatment arm.

### Figures

- Trial flowchart (a Consolidated Standards of Reporting Trials [CONSORT]-style flow diagram to illustrate patient progression through the trial from initial screening for eligibility to completion of the final primary outcome assessment)
- Cumulative incidence function of distant intracranial failure (competing with death) stratified by treatment arm
- Cumulative incidence function of local intracranial failure (competing with both death or distant failure as local failure is captured only before distant failure) stratified by treatment arm
- Cumulative incidence function overall (first distant or local) intracranial failure (competing with death) stratified by treatment arm
- Cumulative incidence function of melanoma death (competing with other cause of death) stratified by treatment arm
- Cumulative incidence function deterioration in cognitive performance (competing with death) stratified by treatment arm
- Cumulative incidence function of melanoma death (competing with non-melanoma death) stratified by treatment arm
- Cumulative incidence function of intracranial death (competing with non-intracranial death) stratified by treatment arm
- Cumulative incidence function of deterioration in role function (competing with death)
- Cumulative incidence function of global QoL (competing with death)
- Cumulative incidence function of drowsiness (competing with death)
- Cumulative incidence function of communication difficulties (competing with death)
- Cumulative incidence function of motor dysfunction (competing with death)
- Cumulative incidence function of social function items/domains (competing with death)
- Evolution of Hopkins Verbal Learning Test-Revised, Delayed Recall (HVLT-R DR) and Controlled Oral Word Association Test stratified by treatment arm
- Evolution of HVLT-R, Total Recall and Delayed Recognition stratified by treatment arm
- Evolution of Controlled Oral Word Association Test stratified by treatment arm
- Evolution of Trail Making Test Parts A and B stratified by treatment arm
- Evolution of Stroop Color and Word Test (Adult Version) stratified by treatment arm
- Evolution of Digit Span (Forward and Backward) stratified by treatment arm
- Kaplan–Meier overall survival curves by treatment arm
- Cumulative incidence of local intracranial failure (competing with distant intracranial failure and death) stratified by Observation vs. HA-WBRT vs. WBRT
- Cumulative incidence of time to distant intracranial failure stratified by Observation vs. HA-WBRT vs. WBRT
- Kaplan–Meier overall survival stratified by Observation vs. HA-WBRT vs. WBRT
- Evolution of HVLT-R DR stratified by Observation vs. HA-WBRT vs. WBRT
- Evolution of HVLT-R, Total Recall and Delayed Recognition stratified by Observation vs. HA-WBRT vs. WBRT
- Kaplan–Meier time to global cognitive deterioration – HVLT-R
- Likert scale for EQ-5D-5 L dimensions/items
- Likert scale for EORTC QLQ-C30 dimensions/items
- Likert scale for EORTC QLQ-BN20 dimensions/items

## Abbreviations

ANZMTG: Australia and New Zealand Melanoma Trials Group
eGFR: Estimated glomerular filtration rate
EORTC: European Organisation for Research and Treatment of Cancer
GEE: Generalised estimating equation
Gy: Gray (unit of radiation)
HVLT-R: Hopkins Verbal Learning Test-Revised
MLM: Mixed linear model
MRI: Magnetic resonance imaging
NCF: Neurocognitive function
QoL: Quality of life
RCI: Reliable Change Index
SRS: Stereotactic radiosurgery
TROG: Trans-Tasman Radiation Oncology Group
WBRT: Whole brain radio therapy
